# Augmenting *Leucaena leucocephala* biomass with mineral fertiliser on rainwater use efficiency, agronomic efficiency and yields on sorghum (*Sorghum bicolor* [(L.) Moench]) under rainwater harvesting techniques in semi-arid region of Zimbabwe

**DOI:** 10.1016/j.heliyon.2022.e09826

**Published:** 2022-06-28

**Authors:** Andrew Tapiwa Kugedera, George Nyamadzawo, Ronald Mandumbu

**Affiliations:** a Department of Environmental Science, Bindura University of Science Education, P. Bag, 1020, Bindura, Zimbabwe; b Department of Agribusiness and Continuing Education, University of Zimbabwe, P.O. Box, MP167, Mount Pleasant Harare, Zimbabwe; c Department of Crop Science, Bindura University of Science Education, P. Bag, 1020, Bindura, Zimbabwe

**Keywords:** Rainwater harvesting, *L. leucocephala*, NPK fertiliser, *Sorghum*, Semi-arid regions, Sandy soils

## Abstract

Food security in semi-arid regions is threatened by declining soil fertility, soil moisture stress and long frequent droughts as a result of erratic rainfall. Therefore we assessed the effects of augmenting *Leucaena leucocephala* biomass (organic manure) with mineral fertiliser on rainwater use efficiency, agronomic efficiency, grain and stover yields for two sorghum varieties (Macia and SV1) under rainwater harvesting techniques. The experiment was laid in split-split plot arrangement with rainwater harvesting method as main plot factor, with *Leucaena* biomass + NPK fertiliser as subplot factor and sorghum variety as sub-sub plot factor over three cropping seasons. Data collected include rainwater use efficiency, agronomic efficiency, grain and stover yields. Results show that tied contours have significantly (p ≤ 0.05) higher grain and stover yields from all varieties and seasons. Irregardless of sorghum variety, tied contours had significant (p ≤ 0.05) sorghum grain and stover yield followed by infiltration pits and lastly standard contours. Grain and stover yields improved with increasing levels of *Leucaena* biomass + NPK fertiliser combination. Highest grain yields observed were 1.146 t ha^−1^ (Macia) and 1.1 t ha^−1^ (SV1) from tied contour +15 t ha^−1^ biomass +150 kg ha^−1^ NPK fertiliser treatments. Rainwater use efficiency was significantly (p ≤ 0.05) higher from tied contours compared to infiltration pits and standard contour. Rainwater use efficiency was statistically (p ≤ 0.05) influenced by increasing application levels of *Leucaena* biomass + NPK fertiliser in all seasons. Agronomic efficiencies were considerably (p ≤ 0.05) affected by rainwater harvesting, *Leucaena* biomass + NPK fertiliser and interaction of all factors. It can therefore be concluded that *Leucaena* biomass + NPK fertiliser, tied contours and infiltration pits improve sorghum yields. Augmenting 2.5 t ha^−1^ biomass with 25 kg ha^−1^ NPK fertiliser under tied contours and Macia have better agronomic efficiency.

## 1 Introduction

Nutrient management is a key factor under rainfed agriculture to improve food security in sub-Saharan Africa (SSA) because agricultural production is failing to meet food demands (Gram, 2020). Small holdings in semi-arid regions are mainly affected by soil degradation which causes soil infertility. This causes small holdings in semi-arid regions to suffer on low nutrient status and efficiencies especially nitrogen (N) which is the most limiting nutrient (Cobo et al., 2010; Tully et al., 2015; Zingore et al., 2015; Gram et al., 2020). To increase efficiency of rainfed agriculture in meeting food demand, there is need for increasing concentration in agricultural systems. Sustainable nutrient intensification is a key aspect in reducing food insecurity and soil degradation. Many farmers in SSA have been applying inadequate organic and inorganic fertilisers, failing to meet required levels needed in improving soil nutrient status and increasing crop yields (Mucheru-Muna et al., 2014; Murimi et al., 2020).

Continuous conventional cultivation and monoculture have resulted in soil degradation causing loss of soil organic matter, increased soil erosion and reduced crop yields. Application of organic manure such as *Leucaena leucocephala* biomass has the capacity to increase soil organic matter, microbial population and improve soil structure (Mafongoya and Dzowela, 1999). Organic nutrient sources can be augmented with mineral fertiliser at reduced rates to reduce soil acidity and increase nutrient availability. The use of organic nutrient sources significantly improves soil organic matter (SOM), soil structure (Vanlauwe et al., 2014) and release nutrient slowly in the plant root zone hence increase nutrient availability to crops (Muchai et al., 2020). By contrast, inorganic nutrient sources have the capacity to quickly release nutrients and maintain positive nutrient balances in soil thereby increasing crop growth and yields (Hakeem et al., 2018). The use of organic and inorganic nutrient sources have been reported to increase sorghum grain and stover yields (Mahinda et al., 2018).

Climate change is another factor which negatively affected crop production in SSA (Nyagumbo et al., 2019). Rainfall variability is increasing rapidly in many semi-arid areas of Africa, especially marginalised areas of SSA where sorghum is a staple food. *Sorghum* is grown as rainfed crop in areas characterised with low and erratic rainfall (Kilasara et al., 2015; Hakeem et al., 2018; Masaka et al., 2019). Performance of sorghum is mainly affected by soil moisture stress and declining soil fertility causing low grain yields (Masaka et al., 2019). *Sorghum* grain yields have been declining in most marginalised areas due to low soil fertility amendments for example in Zimbabwe grain yield averages at 532 kg ha^−1^ (Chiduza et al., 1995; Twomlow et al., 2008). There is need to adopt soil fertility management options to improve nutrient availability in the plant root zone and increase crop yields (Njeru et al., 2013; Vanlauwe et al., 2014) particularly sorghum in semi-arid regions. However, soil moisture in semi-arid areas also contribute to low sorghum yields hence the need to adopt rainwater harvesting techniques to improve soil moisture (Zougmoré et al., 2004; Ayanlade et al., 2018; Muchai et al., 2020). Integration of organic and inorganic nutrient sources with RWH techniques can improve soil nutrients status, water retention and grain yields (Zougmoré et al., 2003, 2014; Rockstrom et al., 2010). Several authors (e.g. Nyakudya et al., 2014; Kilasara et al., 2015; Nyamadzawo et al., 2015; Nyagumbo et al., 2019; Chilagane et al., 2020; Mandumbu et al., 2021) reported that rainwater harvesting techniques reduced surface runoff, harvest a lot of rainfall, store it and sustain plant during dry spell. This combination brings in climate smart agriculture which has the potential to improve sorghum production in semi-arid regions. Therefore we assessed the effects of augmenting *Leucaena leucocephala* biomass with mineral fertiliser on rainwater use efficiency, agronomic efficiency, grain and stover yields for two sorghum varieties (Macia and SV1) under rainwater harvesting techniques.

## 2 Materials and methods

### 2.1 Study site

The study was carried out as on-farm experiment in ward 11 (20º13.441՛ S and 30º28.656՛ E, 775 m above sea level) of Chivi District, which is located at 78 km west of Masvingo in southeastern part of Zimbabwe. The area is located in agroecological region V which is characterised by monomodal rainfall with a 30 year average of 335 mm per season (Mugandani et al., 2012; Mugandani and Mafongoya, 2019). The experimental site is characterised with short growing season associated with frequent mid-season droughts and mean annual temperature ranges from 27–32 °C. The soils are loamy sands which are inherently infertile with deficient in nitrogen. Major crops grown include maize (*Zea mays*), sorghum (*Sorghum bicolor*) and groundnuts (*Arachis hypogea*).

### 2.2 Soil sampling and analysis

Fifteen soil samples were collected from experimental site before planting at a depth of 0–40 cm. A composite soil sample (1 kg) was produced by mixing all samples thoroughly. The sample dried on shade, grounded and sieved using 2 mm sieve. The Bouyoucos hydrometer method was used to determine soil texture (Henderson and Bui, 2002) and total nitrogen was determined using Kjeldahl method (Cottenie, 1980). Calcium chloride (CaCl_2_) method was used to determine soil pH. Soil organic carbon (SOC) and available phosphorous were determined using wet digestion and Oslen method respectively (Olsen, 1954).

### 2.3 Experimental design and treatments

A completely randomised block design was used with treatments arranged in split-split plot. Rainwater harvesting methods were used as main treatment factor at three levels (infiltration pits, tied contour and standard contour) with *Leucaena* biomass + NPK fertiliser used as sub-plot factor at five application rates (0, 2.5 + 25, 5 + 50, 10 + 100 and 15 + 150 i.e. *Leucaena* biomass (t ha^−1^) + NPK fertiliser (kg ha^−1^)) and two sorghum varieties (Macia and SV1) were tested. Treatments were replicated three times and repeated from 2017/18 to 2019/20 cropping seasons. Tied contours used were constructed using cross ties after every 5 m producing structures which were 5 m in length × 0.5 m breath × 1 m deep. Cross tie were constructed by leaving soil between each contour. Infiltration pits (IP) were constructed along the contour and measured 3 m in length × 0.5 m breath × 1 m deep with spacing of 0.5 m in each contour. Standard contour of 35 m long was a control treatment. Rainwater harvesting techniques were spaced at 1.5 m. Every sub plot factor measured 20 m long and 5 m wide, replicated three times. Two rows at edge of every plot were used as buffer lines. Sub-sub plot factor were measuring 2 m long × 4.5 m wide and replicated three times. The plots were on an average slope of 3%.

Land preparation was followed by ploughing using animal drawn mouldboard plough to a depth between 15 cm and20cm. Ploughing was done between the block and did not interfere with rainwater harvesting methods. *Leucaena* biomass was incorporated at a rate of 0, 2.5, 5, 10 and 15 t ha^−1^ and NPK fertiliser at a rate of 0, 25, 50, 100 and 150 kg ha^−1^ mixed together. *Leucaena* biomass used was obtained from 5 year old trees which contained 3.5% nitrogen, 0.2% P_2_O_5_, 0.42% magnesium, 1.35% calcium and 1.8% K_2_O. NPK fertiliser used contains 7% N, 14% P_2_O_5_ and 7% K_2_O. The recommended application rates of NPK fertiliser is 350 kg ha^−1^. Planting was done on 20 December 2017, 12 December 2018 and 30 December 2019 for 2017/2018, 2018/2019 and 2019/2020 seasons respectively. *Sorghum* seeds were sown using spacing of 0.75 m between rows and 0.2 m within rows achieving plant population of 66666 plants ha^−1^. Weeding was done twice using hand hoe. Top dressing of sorghum plants was done using using ammonium nitrate fertiliser (34.5% N) at a rate of 150 kg ha^−1^. Split application was done at a rate of 26 kg N ha^−1^ per split, 3 and 7 weeks after emergence. Fall armyworm was controlled using Demise 65EC during vegetative and prior flowering stage.

### 2.4 *Sorghum* varieties

The experiment used two sorghum varieties (Macia and SV1) as test crop. Macia is an open pollinated variety which is widely grown in Chivi due to its drought tolerance and it thrive well under harsh conditions. Macia variety attains its physiological maturity with an average of 115–120 days. The variety has a yield potential of 3–6 t ha^−1^ under favourable conditions (Gasura et al., 2015). SV1 is an open pollinated semi-dwarf variety with an average of 115–125 days to maturity. The variety was developed in Zimbabwe in 1985 (Chikobvu, 2008). Yield potential of SV1 ranges between 3-6 t ha^−1^ under optimum condition. The variety has not been widely grown in Chivi and was used to compare its performance with Macia.

### 2.5 Data collection

#### 2.5.1 Rainfall

Rainfall was measured using a standard rain gauge installed in the experimental site throughout the growing seasons. Rainfall data was collected every morning at 5 am and recorded in the book. Monthly rainfall was calculated by summing up all daily values. Days dry per month were determined.

#### 2.5.2 Grain and stover yield

Grain and stover yields were harvested from 9 m^2^ per treatment plot. Panicles were hand cut using sharp knife and stover was cut closer to the ground. Heads were sun dried, threshed and grain moisture tested using moisture meter. Grains with moisture content of 13.5% were winnowed, weighed and weight extrapolated to tonnes per hectare. Grain yield obtained was adjusted to 13.5% grain moisture content using the following formula:

 Adjusted yield = Actual yield × $\frac{\left(100-A\right)}{\left(100-U\right)}$ (Gram et al., 2020)Where A is the grain moisture content and U means moisture content of 13.5%.

#### 2.5.3 Rain water use efficiency (RWUE)

This is the efficiency in which rainfall is converted to grain, calculated after harvesting of grain using formula by GRDC (2009).

 RWUE (kg Grain mm^−1^ rainfall) = $\frac{Total\;grain\;yield\;\left(kg\;ha^{-1}\right)}{Total\;rainfall\;\left(mm\right)}$ (GRDC, 2009).

#### 2.5.4 Agronomic efficiency (AE)

The agronomic efficiency was calculated as crop yield increase from control treatment divided by amount of amendment applied as given below:

Agronomic efficiency (AE) = $\frac{\text{Grain ​yield ​of ​fertilised ​plot ​}\left(\text{kg}\right)-\text{grain ​yield ​in ​control ​plot}\left(\text{kg}\right)}{Amount\;of\;ammendment\;applied\;\left(kg\right)}$ (Gram et al., 2020).

### 2.6 Statistical data analysis

Data was subjected to two way analysis of variance (ANOVA) for split-plot analysis using Genstat 14th edition. The least significance differences (LSD) was used to identify and separate significant means at 5% levels. Regression analysis was done use Microsoft Excel to estimate correlation coefficient (R^2^).

## 3 Results

### 3.1 Rainfall

Rainfall was recorded using a standard rain gauge installed at the experimental site. Rainfall totals received over three experimental seasons were ranging from 295–305 mm per cropping season with highest (305 mm) received during 2017/18 and 2019/20 seasons (Table 1). The least rainfall was received in 2018/19 cropping season. The experimental area experienced the longest long dry spell (24 days) in 2018/19 cropping season from mid-March to early April 2019. The seasonal rainfalls received in each of the season (305, 295 and 305 mm respectively) were below a 30 year average of 335 mm received in Chivi during the growing season (Table 1). Results in Table 1 show that climate change affected the experimental site, evidenced by a shift in growing season.

**Table 1:** Monthly rainfall received (mm) during a three year experiment.

Season	Month						
	October	November	December	January	February	March	April
2017/18	0	42	65	78	60	48	12
2018/19	0	25	60	80	68	52	10
2019/20	0	30	95	88	54	38	0
Dry days (Average)	30	23	19	18	20	17	25
Rainfall (30 year)	10	28	69	80	70	51	27
Dry days (30 years)	25	22	16	14	15	13	17

### 3.2 Soil characterisation

Soil texture was classified as sandy loam soil with 76% sand, 21% silt and 3% clay. The soil was slightly acidic (pH = 5.8) with 0.02% total nitrogen and 0.27% SOC. Exchangeable cations were 0.11 cmol_c_ kg^−1^ sodium, 0.15 cmol_c_ kg^−1^ potassium, 0.68 cmol_c_ kg^−1^ magnesium and 1.25 cmol_c_ kg^−1^ calcium during the 2017/18 cropping season before planting (Table 2). An increase in all other soil parameters was observed except for sodium which decreased with application of *Leucaena* biomass + NPK fertiliser (Table 2). The residual effect of *Leucaena* biomass has contributed towards increase in total nitrogen, pH and exchangeable cations.

**Table 2:** Physiochemical characteristics of soil from experimental field.

Soil parameter	Composition over three seasons		
	2017/18	2018/19	2019/20
pH (CaCl_2_)	5.8	6.1	6.1
SOC (%)	0.27	0.72	0.84
Total Nitrogen (%)	0.02	0.02	0.023
P_2_O_5_ mg kg^−1^	3.46	4.87	4.92
K_2_O g cmol_c_ kg^−1^	0.15	0.19	0.2
Calcium cmol_c_ kg^−1^	1.25	1.32	1.35
Magnesium cmol_c_ kg^−1^	0.68	0.69	0.71
Sodium cmol_c_ kg^−1^	0.11	0.082	0.056

### 3.3 Effects of RWH techniques on sorghum grain and stover yields

Rainwater harvesting techniques significantly (p ≤ 0.05) affected sorghum grain yields for both varieties, with the trend tied contours having the greatest yield (p ≤ 0.05) followed by infiltration pits and lastly the standard contour (Table 3). The same trend was observed on stover yields from both varieties across all the three seasons. The highest mean grain yield of 0.876 t ha^−1^ and a lowest (0.743 t ha^−1^) were observed over a period of three years. Stover yields ranged from 2.482 t ha^−1^ and the lowest was 2.305 t ha^−1^ observed from the standard contour. Mean stover yield was highest (2.447 t ha^−1^) and a lowest (2.326 t ha^−1^) from tied contours and standard contours over three years respectively (Table 3).

**Table 3:** Effects of RWH on sorghum grain and stover yields.

RWH techniques	Mean Macia grain yield (t ha^−1^)				Mean SV1 grain yield (t ha^−1^)			
	2018	2019	2020	Mean	2018	2019	2020	Mean
Standard contour	0.806^b^	0.811^b^	0.719^b^	0.779	0.766^b^	0.757^c^	0.705^b^	0.743
Infiltration pits	0.877^ab^	0.86^ab^	0.748^ab^	0.823	0.844^ab^	0.787^b^	0.737^ab^	0.789
Tied contour	0.93^a^	0.911^a^	0.786^a^	0.876	0.891^a^	0.862^a^	0.761^a^	0.838
P-value	*<0.05*	*<0.05*	*<0.05*		*<0.05*	*<0.05*	*<0.05*	
LSD_0.05_	0.0849	0.0521	0.0439		0.0849	0.0521	0.0439	
	**Mean Macia stover yield (t ha^−1^)**				**Mean SV1 stover yield (t ha^−1^)**			
Standard contour	2.32^c^	2.316^a^	2.343^c^	2.326	2.317^c^	2.305^b^	2.394^a^	2.339
Infiltration pits	2.413^b^	2.404^ab^	2.377^b^	2.398	2.402^b^	2.387^ab^	2.328^b^	2.372
Tied contour	2.482^a^	2.461^a^	2.398^a^	2.447	2.481^a^	2.451^a^	2.383^a^	2.438
P-value	*0.023*	*NS*	*<0.05*		*0.023*	*NS*	*<0.05*	
LSD_0.05_	0.0548	0.1048	0.0497		0.0548	0.1048	0.0497	

Same superscripts in same column denotes no significant different between treatments at p ≤ 0.05.

### 3.4 Effects of *Leucaena* biomass + NPK fertiliser on sorghum grain and stover yields

**Figure 1:**
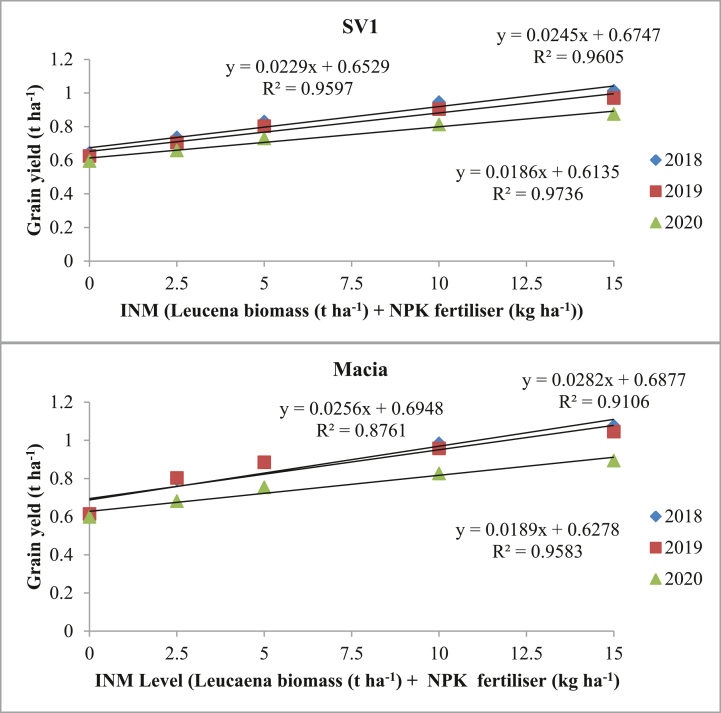
Relationship between Integrated nutrient management and sorghum grain yields. Where: 0 = No amendment; 2.5 = 2.5tha^−1^*Leucaena* biomass+25 kg ha^−1^NPK fertiliser; 5 = 5 t ha^−1^*Leucaena* biomass +50 kg ha^−1^NPK fertiliser; 10 = 10 t ha^−1^*Leucaena* biomass +100 kg ha^−1^NPK fertiliser and 15 = 15 t ha^−1^*Leucaena* biomass +150 kg ha^−1^NPK fertiliser.

*Leucaena* biomass + NPK fertiliser show significant effects (p ≤ 0.05) on grain yields of both Macia and SV1 sorghum varieties. Increasing application rate of *Leucaena* biomass + NPK fertiliser show increased grain yield for both varieties although Macia outperformed SV1 variety. Increasing *Leucaena* biomass + NPK fertiliser levels show increase in three year mean yield (0.61–1.01 t ha^−1^ and 0.62–0.95 t ha^−1^) for Macia and SV1 varieties respectively. Highest grain yield (1.08 t ha^−1^) was obtained from *Leucaena* biomass + NPK fertiliser treatment with 15 t ha^−1^ *Leucaena* biomass +150 kg ha^−1^ NPK fertiliser in 2017/18 season under Macia variety. The relationship between grain yield and *Leucaena* biomass + NPK fertiliser were significantly (p ≤ 0.05) correlated with R^2^ ranging from 0.88-0.96 for Macia and 0.96–0.97 for SV2 (Figure 1). SV1 variety had perfect positive correlation with *Leucaena* biomass + NPK fertiliser at different level throughout the three seasons compared to Macia variety. SV1 had a higher (R^2^ = 0.97) correlation in 2019/20 season compared to all other seasons and Macia variety (Figure 1).

**Figure 2:**
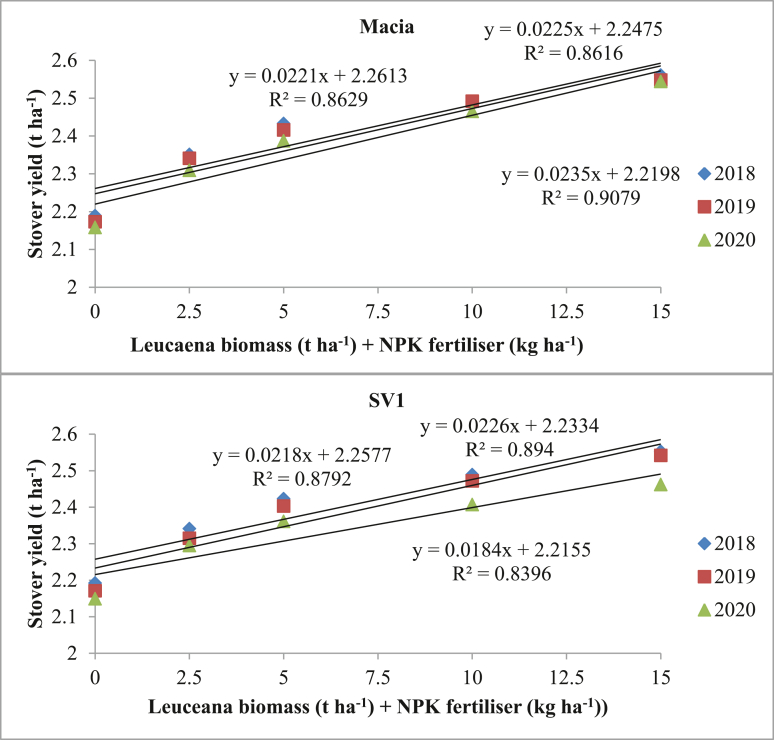
The relationships between stover yields and *Leucaena* biomass + NPK fertiliser quantity. Where: 0 = No amendment; 2.5 = 2.5tha^−1^*Leucaena* biomass+25 kg ha^−1^NPK fertiliser; 5 = 5 t ha^−1^*Leucaena* biomass +50 kg ha^−1^NPK fertiliser; 10 = 10 t ha^−1^*Leucaena* biomass +100 kg ha^−1^NPK fertiliser and 15 = 15 t ha^−1^*Leucaena* biomass +150 kg ha^−1^NPK fertiliser.

Stover yield were statistically affected (p ≤ 0.05) by different *Leucaena* biomass + NPK fertiliser levels except in 2018/19 season where no significant effect (p > 0.05) was observed. Increasing *Leucaena* biomass + NPK fertiliser levels show significant effect on stover yields with highest yield of 2.56 t ha^−1^ observed in 2017/18 season from 15 t ha^−1^ *Leucaena* biomass +150 kg ha^−1^ NPK fertiliser + Macia variety. Regardless of *Leucaena* biomass + NPK fertiliser and season, Macia showed higher stover yields than SV1. The results also show significant correlation (p ≤ 0.05; R^2^ = 0.84–0.91) between different levels of *Leucaena* biomass + NPK fertiliser and stover yield (Figure 2). Macia stover yields were positively correlated (R^2^ = 0.86–0.91) to *Leucaena* biomass + NPK fertiliser levels. The highest correlation (R^2^ = 0.91) was observed in 2019/20 season (Figure 2). Furthermore, results show positive correlation (R^2^ = 0.83–0.89) between SV1 stover yields and *Leucaena* biomass + NPK fertiliser levels. The highest correlation (R^2^ = 0.89) was observed in 2018/19 season (Figure 2).

### 3.5 Interactive effects of RWH, leucaena biomass + NPK fertiliser and variety on sorghum grain yields

Rainwater harvesting practices × *Leucaena* biomass + NPK fertiliser × variety show significant interaction effects (p ≤ 0.05) on sorghum grain yields across all treatments (Table 4). Effects of RWH × *Leucaena* biomass + NPK fertiliser × Macia variety had yield advantage compared to SV1 variety. The highest grain yield (1.146 t ha^−1^) was obtained from tied contour +15 t ha^−1^ *Leucaena* biomass +150 kg ha^−1^ NPK fertiliser + Macia variety during 2017/18 cropping season. Tied contour × *Leucaena* biomass + NPK fertiliser × Macia variety had the highest grain yields (0.612–1.146 t ha^−1^) followed by infiltration pits (0.604–1.104 t ha^−1^) throughout the three cropping seasons. *Sorghum* variety SV1 had yields ranging from 0.609–1.1 t ha^−1^ (tied contour × *Leucaena* biomass + NPK fertiliser) and 0.595–1.021 t ha^−1^ (infiltration pits × *Leucaena* biomass + NPK fertiliser). However, standard contour × *Leucaena* biomass + NPK fertiliser had the lowest grain yields ranging from 0.571–0.989 t ha^−1^ (Macia) and 0.581–917 t ha^−1^ (SV1) compared to other RWH practices × *Leucaena* biomass + NPK fertiliser levels (Table 4).

**Table 4:** Interaction of RWH, *Leucaena* biomass + NPK fertiliser and variety on sorghum grain yields.

Treatments	Macia Mean grain yield (kg ha^−1^)				SV1 Mean grain yield (kg ha^−1^)		
RWH techniques	Leucaena biomass t ha^−1^ + NPK fertiliser kg ha^−1^	2018	2019	2020	2018	2019	2020
Infiltration pits	0	0.636^h^	0.636^g^	0.604^i^	0.631^j^	0.606^g^	0.595^g^
	2.5/25	0.807^f^	0.817^e^	0.664^h^	0.752^h^	0.675^f^	0.653^ef^
	5/50	0.89^e^	0.882^d^	0.758^f^	0.836^g^	0.787^e^	0.752^d^
	10/100	0.947^d^	0.959^c^	0.822^d^	0.978^c^	0.883^cd^	0.812^c^
	15/150	1.104^b^	1.011^b^	0.89^b^	1.021^b^	0.982^b^	0.872^b^
Tied contour	0	0.634^h^	0.636^g^	0.612^a^	0.699^i^	0.671^f^	0.609^e^
	2.5/25	0.823f	0.813^e^	0.732^f^	0.776^h^	0.772^f^	0.684^e^
	5/50	0.952^d^	0.959^c^	0.794^g^	0.884^e^	0.867^d^	0.749^d^
	10/100	1.095^c^	1.011^b^	0.861^c^	0.998^c^	0.97^b^	0.856^bc^
	15/150	1.146^a^	1.137^a^	0.929^a^	1.1^a^	1.03^a^	0.905^a^
Standard contour	0	0.574^i^	0.571^h^	0.58^ij^	0.602^jk^	0.6^g^	0.581^g^
	2.5/25	0.754^g^	0.779^f^	0.648^hi^	0.678^i^	0.672^f^	0.636.7^f^
	5/50	0.813^f^	0.813^e^	0.709^g^	0.771^h^	0.754^e^	0.688^e^
	10/100	0.911^e^	0.904^d^	0.796^e^	0.861^f^	0.86^d^	0.77^d^
	15/150	0.977^d^	0.989^b^	0.863^c^	0.917^d^	0.899^c^	0.849^bc^
P-value		*<0.001*	*<0.001*	*<0.001*	*<0.001*	*<0.001*	*<0.001*
LSD_0.05_		*0.019*	*0.0117*	*0.00981*	*0.019*	0.0117	0.00981
CV (%)		1.4	0.9	0.8	*1.4*	*0.9*	*0.8*

Same superscripts in same column denotes no significant different between treatments at p ≤ 0.05.

### 3.6 Interaction effects of RWH, leucaena biomass + NPK fertiliser and variety on sorghum stover yields

Rainwater harvesting techniques × *Leucaena* biomass + NPK fertiliser × sorghum variety had significant interaction effect (p ≤ 0.05) on stover yield throughout three cropping seasons. Regardless of RWH and season, stover yields from Macia were greater than those obtained from SV1 at each application rate of *Leucaena* biomass + NPK fertiliser (Table 5). Stover yields from Macia variety at each RWH technique and different levels of *Leucaena* biomass + NPK fertiliser ranged of 2.09–2.667 t ha^−1^ compared to 2.1–2.666 t ha^−1^ from SV1variety across all treatment combinations. Tied contour and infiltration pits integrated with different *Leucaena* biomass + NPK fertiliser levels show higher yields compared to standard contour which had lowest stover yields throughout the three seasons (Table 5).

**Table 5:** Interaction of RWH, Leucaena biomass + NPK fertiliser and sorghum variety on stover yields.

RWH techniques	Leucaena biomass t ha^−1^ + NPK fertiliser kg ha^−1^	Macia Mean stover yield (tha^−1^)			SV1Mean stover yield (tha^−1^)		
		2018	2019	2020	2018	2019	2020
Infiltration pits	0	2.193^h^	2.184^i^	2.183^g^	2.192^g^	2.172^h^	2.167^g^
	2.5/25	2.377^f^	2.382^e^	2.296^f^	2.353^e^	2.33^ef^	2.269^f^
	5/50	2.464^cd^	2.436^d^	2.41^d^	2.438^cd^	2.402^d^	2.354^d^
	10/100	2.478^c^	2.503^c^	2.456^c^	2.479^c^	2.488^c^	2.398^c^
	15/150	2.552^b^	2.515^j^	2.538^j^	2.547^b^	2.543^b^	2.452^ab^
Tied contour	0	2.284^c^	2.219^h^	2.188^g^	2.286^f^	2.225^g^	2.181^g^
	2.5/25	2.398^ef^	2.366^e^	2.356^ef^	2.394^d^	2.37^e^	2.369^d^
	5/50	2.484^c^	2.494^c^	2.419^d^	2.482^c^	2.477^c^	2.411^bc^
	10/100	2.578^b^	2.567^b^	2.477^b^	2.579^b^	2.558^b^	2.46^ab^
	15/150	2.667^a^	2.658^a^	2.551^a^	2.666^a^	2.624^a^	2.494^a^
Standard contour	0	2.09^i^	2.115^j^	2.102^h^	2.1^h^	2.116^i^	2.1^h^
	2.5/25	2.278^g^	2.274^g^	2.275^f^	2.277^f^	2.246^g^	2.247^f^
	5/50	2.35^f^	2.317^f^	2.332^ef^	2.35^e^	2.331^ef^	2.319^de^
	10/100	2.415^e^	2.406^de^	2.462^c^	2.411^d^	2.37^e^	2.365^d^
	15/150	2.465^cd^	2.472^cd^	2.543^a^	2.447^cd^	2.459^c^	2.439^b^
P-value		*0.03*	*<0.001*	*<0.001*	*0.03*	*<0.001*	*<0.001*
LSD_0.05_		0.0123	0.0234	0.0111	0.0123	0.0234	0.0111
CV (%)		*0.3*	*0.6*	*0.3*	*0.3*	*0.6*	*0.3*

### 3.7 Rainwater use efficiency (RWUE)

**Figure 3:**
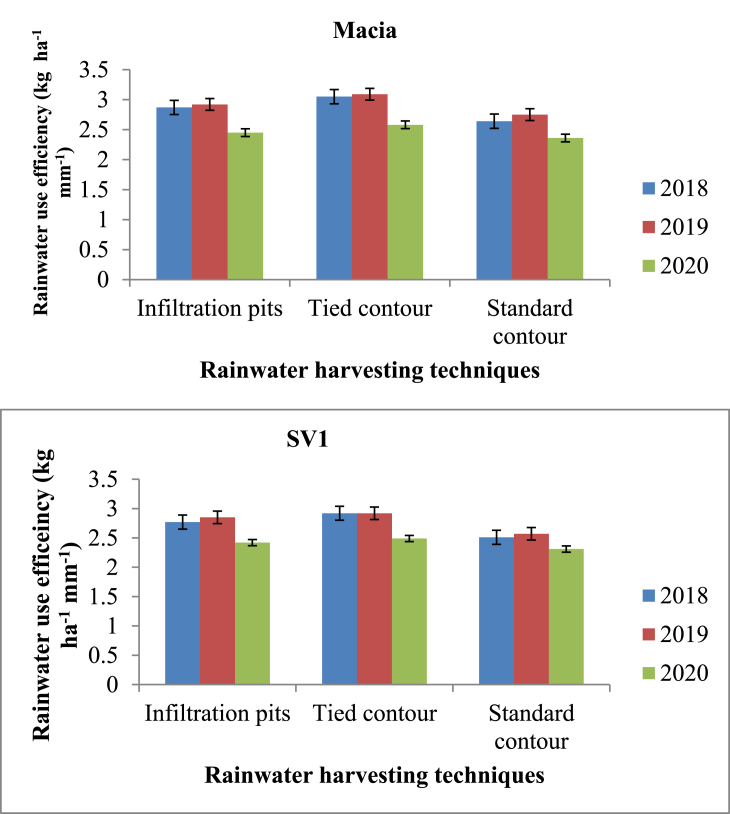
Effect of rainwater harvesting techniques on rainwater use efficiency of two sorghum varieties.

Rainwater harvesting methods statistically (p ≤ 0.05) affected RWUE in all seasons. Effects of RWH techniques were significant on RWUE for both varieties except in 2017/18 season where no significant effect was observed (p > 0.05). Macia variety had higher RWUE which ranged from 2.58–2.91 kg ha^−1^ mm^−1^ compared to SV1 (2.48–2.78 kg ha^−1^ mm^−1^) over three years (Figure 3). Tied contour recorded highest RWUE (3.09 kg ha^−1^ mm^−1^) from Macia variety in 2018/19 season and a lowest of 2.31 kg ha^−1^ mm^−1^ from standard contour + SV1 in 2019/20 season. Results also show significant differences (p ≤ 0.05) among varieties. Standard contour treatments had the lowest RWUE regardless of variety and season, with RWUE ranging from 2.31–2.75 kg ha^−1^ mm^−1^ (Figure 3).

**Figure 4:**
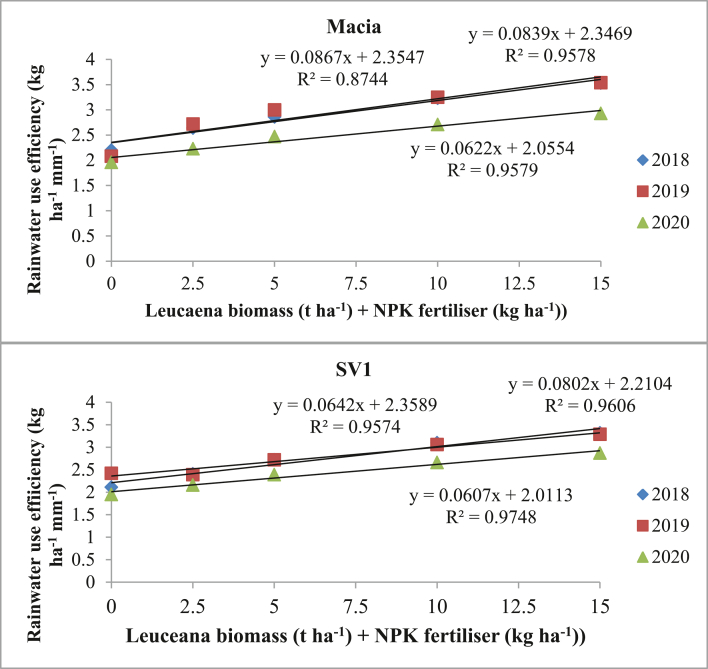
The relationships between RWUE and *Leucaena* biomass + NPK fertiliser levels. Where: 0 = No amendment; 2.5 = 2.5tha^−1^*Leucaena* biomass+25 kg ha^−1^NPK fertiliser; 5 = 5 t ha^−1^*Leucaena* biomass +50 kg ha^−1^NPK fertiliser; 10 = 10 t ha^−1^*Leucaena* biomass +100 kg ha^−1^NPK fertiliser and 15 = 15 t ha^−1^*Leucaena* biomass +150 kg ha^−1^NPK fertiliser.

RWUE was considerably affected (p ≤ 0.05) by *Leucaena* biomass + NPK fertiliser levels regardless of sorghum variety and season. Increasing *Leucaena* biomass + NPK fertiliser levels over control show significant increase in RWUE with highest (3.53 kg ha^−1^ mm^−1^) and lowest of 2.02 kg ha^−1^ mm^−1^ observed from Macia variety. Macia variety responds well from different levels of *Leucaena* biomass + NPK fertiliser, with three year mean ranging from 2.02–3.33 kg ha^−1^ mm^−1^ compared to 2.16–3.16 kg ha^−1^ mm^−1^ from SV1 variety. RWUE from Macia was positively correlated to *Leucaena*/NPK fertiliser levels but less than that of SV1 (Figure 4). SV1 variety was highly correlated (R^2^ = 0.96–0.97) to *Leucaena* biomass + NPK fertiliser application rates. The highest correlation (R^2^ = 0.97) was observed in 2019/20 cropping season from SV1 variety (Figure 4). Macia variety had the lowest correlation with increase in *Leucaena* biomass + NPK fertiliser levels in 2017/18 cropping season.

Rainwater use efficiency was statistically affected (p ≤ 0.05) by interaction of RWH, *Leucaena* biomass + NPK fertiliser and sorghum variety (Table 6). Highest RWUE (3.85 kg ha^−1^ mm^−1^) was obtained in 2018/19 from tied contour +15 t ha^−1^ *Leucaena* biomass +150 kg ha^−1^ NPK fertiliser treatment + Macia variety. Furthermore, lowest RWUE (1.88 kg ha^−1^ mm^−1^) was observed from SC with no amendment under Macia variety (Table 6). Tied contour and IP show better RWUE compared to standard contours which had lowest RWUE throughout three cropping seasons.

**Table 6:** Effects of RWH, Leucaena biomass + NPK fertiliser and variety on RWUE.

Treatments		Mean Macia RWUE (kg ha^−1^ mm^−1^)			Mean SV1 RWUE (kg ha^−1^ mm^−1^)		
RWH techniques	Leucaena biomass t ha^−1^ + NPK fertiliser kg ha^−1^	2018	2019	2020	2018	2019	2020
Infiltration pits	0	2.08^f^	2.15^f^	1.98^i^	2.07^h^	2.95^c^	1.95^gh^
	2.5/25	2.63^d^	2.77^e^	2.18^h^	2.47^f^	2.29^e^	2.14^f^
	5/50	2.92^c^	2.99^d^	2.48^f^	2.74^e^	2.67^d^	2.46^de^
	10/100	3.1^bc^	3.25^c^	2.7^d^	3.21^c^	2.99^c^	2.66^cd^
	15/150	3.62^a^	3.43^b^	2.92^b^	3.35^b^	3.33^b^	2.86^b^
Tied contour	0	2.08^f^	2.16^f^	2.01^i^	2.29^g^	2.27^e^	2^g^
	2.5/25	2.7^d^	2.75^e^	2.4^f^	2.54^f^	2.62^d^	2.24^f^
	5/50	3.12^bc^	3.25^c^	2.6^e^	2.9^d^	2.94^c^	2.46^de^
	10/100	3.59^ab^	3.43^b^	2.82^c^	3.27^b^	3.29^b^	2.81^b^
	15/150	3.76^a^	3.85^a^	3.05^a^	3.61^a^	3.49^a^	2.99^a^
Standard contour	0	1.88^g^	1.94^g^	1.9^i^	1.97^h^	2.03^f^	1.9^gh^
	2.5/25	2.57^de^	2.64^d^	2.12^h^	2.22^g^	2.28^e^	2.09^g^
	5/50	2.55^de^	2.75^e^	2.33^g^	2.53^f^	2.56^d^	2.26^f^
	10/100	2.99^c^	3.06^d^	2.61^e^	2.82^e^	2.92^c^	2.53^d^
	15/150	3.2^bc^	3.35^bc^	2.83^c^	3.01^d^	3.05^c^	2.78^bc^
P-value		*<0.001*	*<0.001*	*<0.001*	*<0.001*	*<0.001*	*<0.001*
LSD_0.05_		*0.0623*	*0.0393*	*0.0323*	0.0623	0.0393	0.0323
CV (%)		1.4	0.8	0.8	*1.4*	*0.8*	*0.8*

Same superscripts in same column denotes no significant different between treatments at p ≤ 0.05.

### 3.8 Agronomic efficiency of sorghum under RWH techniques and *Leucaena*/NPK fertiliser

Higher sorghum agronomic efficiency (AE) of 0.054 kg kg^−1^ was obtained in 2017/18 season from Macia grown under tied contours. *Sorghum* agronomic efficiency was considerably affected (p ≤ 0.05) by RWH methods (Table 7). On average, sorghum AE was better from Macia under tied contours compared to same treatment under SV1 variety across all seasons.

**Table 7:** Influence of RWH on Agronomic efficiency of sorghum.

RWH techniques	Mean Macia AE (kg kg^−1^)				Mean SV1 AE (kg kg^−1^)			
	2018	2019	2020	Mean	2018	2019	2020	Mean
Standard contour	0.044^b^	0.023^b^	0.047^b^	0.038	0.027^c^	0.02^c^	0.026^c^	0.024
Infiltration pits	0.045^b^	0.023^b^	0.044^c^	0.037	0.037^a^	0.023^b^	0.029^b^	0.03
Tied contour	0.054^a^	0.032^a^	0.051^a^	0.046	0.031^b^	0.025^a^	0.033^a^	0.03
*P-value*	*<0.001*	*<0.001*	*<0.001*		*<0.001*	*<0.001*	*<0.001*	
LSD_0.05_	0.0046	0.0016	0.0026		0.0046	0.0016	0.0026	

**Figure 5:**
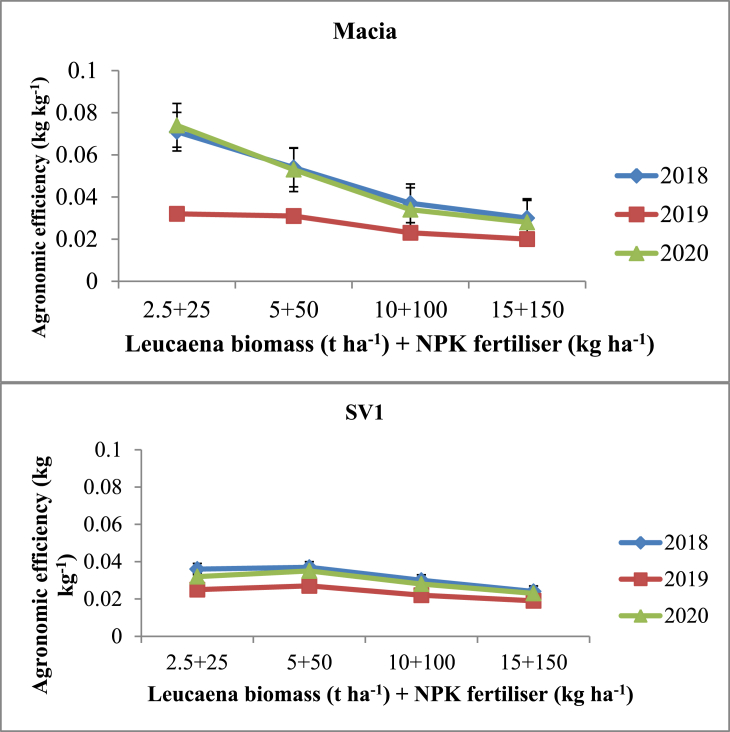
Effects of *Leucaena* biomass + NPK fertiliser on agronomic efficiency (AE) of Macia and SV1.

*Sorghum* agronomic efficiency was statistically influenced (p ≤ 0.05) by different application rates of *Leucaena* biomass + NPK fertiliser. Higher AE of 0.074 kg kg^−1^ was observed from Macia variety +2.5 t ha^−1^ *Leucaena* biomass +25 kg ha^−1^ NPK fertiliser in 2019/20 cropping season (Figure 5). Agronomic efficiency decreased with increasing levels of nutrient sources. Macia variety had better AE compared to SV1 variety at all application rates. Agronomic efficiency of SV1 variety were in the same range as influenced by *Leucaena* biomass + NPK fertiliser × season (Figure 5).

*Sorghum* agronomic efficiency was considerably influenced (p ≤ 0.05) by integration of RWH and *Leucaena* biomass + NPK fertiliser except in 2017/18 season where no significant effect (p > 0.05) on AE was observed (Table 8). Highest sorghum agronomic efficiency of 0.082 kg kg^−1^ was observed from standard contour +2.5 t ha^−1^ *Leucaena* biomass +25 kg ha^−1^ NPK fertiliser. Macia variety show better AE compared to SV1 in all three cropping seasons (Table 8).

**Table 8:** Interaction of RWH and *Leucaena* biomass + NPK fertiliser on Macia and SV1 agronomic efficiency.

Treatments		Mean Macia AE (kg kg^−1^)			Mean SV1 AE (kg kg^−1^)		
Rainwater harvesting techniques	*Leucaena* biomass (t ha^−1^) + NPK fertiliser (kg ha^−1^)	2018	2019	2020	2018	2019	2020
Infiltration pits	2.5/25	0.068	0.022^e^	0.072^b^	0.048	0.023^bc^	0.027^c^
	5/50	0.05	0.03^c^	0.049^c^	0.041	0.031^a^	0.036^ab^
	10/100	0.031	0.022^e^	0.032^d^	0.034	0.021^bc^	0.027^bc^
	15/150	0.031	0.019^f^	0.025^e^	0.026	0.018^i^	0.025^bc^
Tied contour	2.5/25	0.075	0.047^a^	0.07^b^	0.031	0.03^a^	0.04^a^
	5/50	0.063	0.036^b^	0.064^b^	0.037	0.028^a^	0.039^a^
	10/100	0.046	0.025^h^	0.037^d^	0.03	0.024^b^	0.032^b^
	15/150	0.034	0.021^j^	0.033^d^	0.027	0.02^bc^	0.024^bc^
Standard contour	2.5/25	0.07	0.027^c^	0.082^a^	0.03	0.022^bc^	0.029^b^
	5/50	0.047	0.026^d^	0.047^c^	0.033	0.021^bc^	0.031^b^
	10/100	0.033	0.022^e^	0.033^d^	0.026	0.019^bc^	0.026^bc^
	15/150	0.027	0.019^f^	0.028^de^	0.021	0.018^cd^	0.02^c^
*P-value*		*NS*	*<0.001*	*<0.001*	*NS*	*<0.001*	*<0.001*
LSD_0.05_		0.0093	0.0031	0.0052	0.0093	0.0031	0.0052
CV (%)		*14.2*	*7.7*	*8.2*	*14.2*	*7.7*	*8.2*

Same superscripts in same column denotes no significant different between treatments at p ≤ 0.05.

## 4 Discussion

### 4.1 Rainfall

Rainfall received in the experimental site in all seasons was less than the 30 year average of 335 mm and ranged from 295-305 mm per growing season. Rainfall received was influenced by frequent dry spells and cannot sustain crops to harvesting stage. These results were confirming assertion by Mugandani et al. (2012), Mugandani and Mafongoya (2019) and Nyagumbo et al. (2019) who reported that climate change affected rainfall in semi-arid regions. Climate changes greatly affected Chivi district with rainfall received ranges from 295-305 mm per growing season compared to a 30 year average of 335 mm. Dry days were high per month compared to 30 year average due to climate change. This may call for adoption of climate smart agriculture to increase crop yields (Mugandani et al., 2012; Thornton et al., 2014; Winter-Nelson et al., 2016).

### 4.2 Soil characterisation

Soil parameters were increased with application levels of *Leucaena* biomass + NPK fertiliser. This could have been contributed by decomposition of *Leucaena* biomass producing nitrogen, exchangeable cations and regulating soil pH. Potassium and phosphorous supplied by NPK fertiliser also contributed towards increase in these soil parameters. Applications of *Leucaena* biomass have been reported to increase SOC, potassium, phosphorous and pH (Timsina, 2018). Soil organic carbon from this study was in the same range of 0.06–2.3% as reported from Chivi by Mapanda and Mavengahama (2011). Organic nutrient sources amended with inorganic fertilisers play a pivotal role in improving soil fertility as a result of decomposition and mineralisation (Gram et al., 2020; Kimaru-Muchai et al., 2021; Mamuye et al., 2021). Augmenting *Leucaena* biomass with NPK fertiliser has shown the potential in improving soil fertility. This may have been a result of high organic carbon and fast decomposition of organic sources (Kisinyo et al., 2019). During decomposition of organic manure, humic acids are produced which bind to Aluminium and increase availability of basic cation essential for growth to crops.

### 4.3 Grain and stover yield

Rainwater harvesting techniques of tied contours and IP improved sorghum grain yields compared to standard contours (SC) during the experimental period. Tied contours and IP recorded higher yields compared to SC which had the lowest grain yields at all seasons and varieties. This may have been associated to increased soil moisture content by tied contour and infiltration pits which increased water availability leading to higher grain yield. This was in agreement with results from related studies by several authors (e.g. Mupangwa et al., 2006; Nyakudya et al., 2014; Kilasara et al., 2015; Nyamadzawo et al., 2015; Mahinda et al., 2018; Nyagumbo et al., 2019; Chilagane et al., 2020; Mandumbu et al., 2021) who reported improved water retention which results in increasing grain yields. Rainwater harvesting techniques retain water in the plant root zone, improving soil moisture content and grain yields (Mandumbu et al., 2021; Kubiku et al., 2022). Availability of water in the plant root zone influence nutrient availability to plants, improve response of crops to nutrients and increase nutrient use efficiency (Mupangwa et al., 2012; Kilasara et al., 2015; Mahinda et al., 2018; Kugedera and Kokerai, 2019; Chilagane et al., 2020; Mandumbu et al., 2021). This is in agreement to results by Mahinda et al. (2018) and Chilagane et al. (2020) who observed higher sorghum and pearl millet yields after using rainwater harvesting methods respectively. Low water retention in standard contour contributed immensely to low sorghum yields throughout three cropping seasons irrespective of sorghum variety. This concurs with findings by several authors (e.g. Mupangwa et al., 2012; Nyamadzawo et al., 2015; Nyagumbo et al., 2019; Kubiku et al., 2022) who reported low yields from standard contours.

Augmenting *Leucaena* biomass with NPK fertiliser increased sorghum grain yields and can be a better option as integrated soil fertility management. Grain yields were significantly increased with increase in application rates of nutrient sources. Organic nutrient sources such as *Leucaena* biomass have the capacity to increase SOC, microbial population and soil total porosity (Mafongoya and Dzowela, 1999) which when augmented with mineral fertiliser can significantly increase sorghum grain yields. This was in agreement with Kilasara et al. (2015) and Kubiku et al. (2022) who reported increased sorghum yields after combining organic manure and mineral fertiliser. Increasing application rates of *Leucaena* biomass + NPK fertiliser improved soil structure, nutrient status and sorghum yields.

*Sorghum* grain yields were significantly improved by integration of RWH techniques and *Leucaena* biomass + NPK fertiliser at different application rates. Grain yields were high from all treatments of tied contours combined with *Leucaena* biomass + NPK fertiliser. This may be attributed to better water capture by tied contours compared to infiltration pits and standard contours. This corroborates with results by Kubiku et al. (2022) who reported higher yields from tied contours combined with cattle manure + *N fertiliser*. Related researches in Africa show that integration of rainwater harvesting with INM increased sorghum grain yields especially in arid and semi-arid areas (Zougmoré et al., 2003; Kilasara et al., 2015; Kimaru, 2017). Water management and INM research in sorghum by Zougmoré et al. (2003), show grain yield ranging from 0.365–2.627 t ha^−1^ which were in the same range (0.59–1.146 t ha^−1^) with results from this study. Results from this study were in agreement with results by Kilasara et al. (2015) who reported increase in sorghum grain yields (0.313–2.787 t ha^−1^) after using infiltration pits (IP) and cattle manure + NPK fertiliser in Tanzania. This was linked to reduced soil erosion, surface runoff and increased soil moisture in plant root zone (Mupangwa et al., 2012; Kilasara et al., 2015; Nyamadzawo et al., 2015; Nyagumbo et al., 2019; Muchai et al., 2020; Mandumbu et al., 2021) and improved nutrient levels in the top soil due to INM (Sebnie et al., 2020).

*Sorghum* stover yields were significantly affected by RWH techniques. This could have been caused by synergic effects of available soil moisture from tied contours and improved nutrient availability (Nyamadzawo et al., 2015; Nyagumbo et al., 2019; Kimaru-Muchai et al., 2021). Stover yield were improved with increasing integrated nutrient management (INM) levels. This was in agreement with Mesfin et al. (2009), Hakeem et al. (2018) and Sebnie et al. (2020) who reported improved sorghum stover yield with increased application rates of nutrient sources. Stover yield was highly correlated to *Leucaena* biomass + NPK fertiliser application rates. Integration of RWH and *Leucaena* biomass + NPK fertiliser show improvements in sorghum stover yields for both varieties. Highest stover yield observed from tied contours integrated with INM may have been caused by improved soil moisture and nutrients availability in plant rooting zone. This may also have been attributed to better soil-water and nutrient management which improved nutrient use efficiency by crops (Mugwe et al., 2019).

### 4.4 Rainwater use efficiency (RWUE)

Rainwater use efficiency was improved by tied contours and infiltration pits than standard contours. Higher RWUE values were observed from tied contours and infiltration pits than standard contours over three cropping seasons. These results were in agreement to findings by Fatondji et al. (2006) and Coulibaly (2015) who reported ranges from 2.2–3.45 kg ha^−1^ mm^−1^ after using insitu rainwater harvesting method of Zai pits. Results from this study were also corroborated to findings by Itabari (1999) and Chiroma et al. (2008) who reported RWUE values ranging from 1.21–3.75 kg ha^−1^ mm^−1^ from sorghum under tied ridges and 2.83–3.38 kg ha^−1^ mm^−1^ from sorghum grown under Zai pits. Increasing application rates of nutrient sources improved RWUE (Hakeem et al., 2018). However, results from integration of RWH techniques and *Leucaena* biomass + NPK fertiliser from this study were higher than findings by Fatondji et al. (2006) which ranges from 1.8–2.1 kg ha^−1^ mm^−1^ after using Zai pits + manure + straw in pearl millet. Results from this experiment were in the same range with results by Gonda (2015) who observed RWUE values ranging from 0.97–2.84 kg ha^−1^ mm^−1^ after using combination of water management techniques + compost manure + NPK fertiliser applied in pearl millet. The use of soil fertility and water management practices has the potential to improve RWUE with 15–25% (Hartfield et al., 2001).

### 4.5 Agronomic efficiency

Tied contours had better sorghum AE compared to infiltration pits and standard contour. Higher AE was observed from Macia variety than SV1. Highest AE from tied contours was in agreement with findings by Fatondji (2002) who reported better AE under insitu rainwater harvesting of *Zai* pits. Agronomic efficiency of sorghum was high at low application levels of nutrient sources (2.5 t ha^−1^ *Leucaena* biomass +25 kg ha^−1^NPK fertiliser). Findings obtained corroborates with results by Gonda (2015) and Coulibaly (2015) who reported low AE with high application levels of nutrient sources. Furthermore, Vanlauwe et al. (2011) and Gram et al. (2020) reported high AE at low rates of inorganic and organic nutrient sources. *Sorghum* agronomic efficiency declined with increasing application levels of *Leucaena* biomass + NPK fertiliser. This was in agreement with results by Fatondji et al. (2006) who reported low AE form manure application rates more than 1000 kg ha^−1^. This was also affirmed by Gram et al. (2020) who reported decline in AE with application of more than 100 kg N ha^−1^. Macia variety proved to have better agronomic efficiency than SV1 and it is a promising variety to improve food security in semi-arid regions especially in SSA. Integration of RWH and *Leucaena* biomass + NPK fertiliser show significant effect on AE. These results were contrary to findings by Coulibaly (2015) who reported no significant effect on AE after combining rainwater harvesting methods, organic and inorganic fertilisers in Mali.

## 5 Conclusion

Results from this study clearly demonstrated that augmenting *Leucaena* biomass with NPK fertiliser has the potential to improve soil fertility, nutrient availability to crops and sorghum yields. Tied contours and infiltration pits had higher sorghum yields than SC in all cropping seasons. Tied contours and Macia variety had better agronomic efficiency when combined with 2.5 t ha^−1^ *Leucaena* biomass +25 kg ha^−1^ NPK fertiliser. Augmenting *Leucaena* biomass with NPK fertiliser under tied contours and Macia variety proved to increase sorghum yields, rainwater water use efficiency and have better agronomic efficiencies in semi-arid regions. Although 15 tha^-1^ *Leucaena* biomass +150 kg ha^−1^ NPK fertiliser gave higher yields, they have low agronomic efficiency compared to 2.5 t ha^−1^ *Leucaena* biomass +25 kg ha^−1^ NPK fertiliser. We can conclude that tied contours combined with 5 t ha^−1^Leucaena biomass +50 kg ha^−1^ NPK + Macia can be adopted by farmers to reduce food insecurity in semi-arid regions due to higher yield increment compared to other treatments. Therefore smaller quantities of *Leucaena* biomass can be used and the rest turned in to fodder for livestock.

## Declarations

### Author contribution statement

Andrew Tapiwa Kugedera: Conceived and designed the experiments; Performed the experiments; Analyzed and interpreted the data; Wrote the paper.

Ronald Mandumbu, George Nyamadzawo: Conceived and designed the experiments; Contributed reagents, materials, analysis tools or data.

### Funding statement

This research did not receive any specific grant from funding agencies in the public, commercial, or not-for-profit sectors.

### Data availability statement

Data included in article/referenced in the article.

### Declaration of interest's statement

The authors declare no conflict of interest.

### Additional information

No additional information is available for this paper.
